# Cloth face masks to prevent Covid-19 and other respiratory infections^*^

**DOI:** 10.1590/1518-8345.4537.3353

**Published:** 2020-08-10

**Authors:** Magda Milleyde de Sousa Lima, Francisco Marcelo Leandro Cavalcante, Thamires Sales Macêdo, Nelson Miguel Galindo-Neto, Joselany Áfio Caetano, Lívia Moreira Barros

**Affiliations:** 1 Universidade Federal do Ceará, Departamento de Enfermagem, Fortaleza, CE, Brazil.; 2 Scholarship holder at the Conselho Nacional de Desenvolvimento Científico e Tecnológico (CNPq), Brazil.; 3 Universidade Estadual Vale do Acaraú, Centro de Ciências da Saúde, Sobral, CE, Brazil.; 4 Instituto Federal de Educação, Ciência e Tecnologia de Pernambuco, Campus Pesqueira, Pesqueira, PE, Brazil.; 5 Universidade da Integração Internacional da Lusofonia Afro-Brasileira, Curso de Enfermagem, Redenção, CE, Brazil.

**Keywords:** Facial Masks, Coronavirus, Coronavirus Infections, Respiratory Tract Infections, Disease Prevention, Review, Máscaras Faciales, Coronavirus, Infecciones por Coronavirus, Infecciones del Sistema Respiratório, Prevención de Enfermidades, Revisión, Máscaras Faciais, Coronavírus, Infecções por Coronavírus, Infecções Respiratórias, Prevenção de Doenças, Revisão

## Abstract

**Objective:**

to analyze scientific evidence on the efficacy of cloth masks in preventing COVID-19 and other respiratory infections.

**Method:**

integrative literature review based on the following guiding question: What is the efficacy of cloth face masks in absorbing particles that cause respiratory infection? The search was conducted in eight electronic databases, without any restriction in terms of language or period.

**Results:**

low coverage cloth face masks made of 100% cotton, scarf, pillowcase, antimicrobial pillowcase, silk, linen, tea towel, or vacuum bag, present marginal/reasonable protection against particles while high coverage cloth masks provide high protection.

**Conclusion:**

cloth face masks are a preventive measure with moderate efficacy in preventing the dissemination of respiratory infections caused by particles with the same size or smaller than those of SARS-CoV-2. The type of fabric used, number of layers and frequency of washings influence the efficacy of the barrier against droplets.

## Abstract (es)

**Objetivo:**

analizar las evidencias científicas sobre la eficacia de las máscaras de tejido para la prevención de COVID-19 y otras infecciones respiratorias.

**Método:**

revisión integradora de la literatura a partir de la pregunta orientadora: ¿qué eficacia tienen las máscaras de tejido en la absorción de partículas que causan infección respiratoria? La búsqueda fue llevada a cabo en ocho bases de datos electrónicas, sin restricciones de tiempo e idioma.

**Resultados:**

las máscaras de tejido de baja cobertura, 100% algodón, bufanda, funda, funda antimicrobiana, lino, seda, toalla de té y bolsa de aspiradora demuestran una protección marginal/razonable en la absorción de partículas, mientras que las máscaras de tejido de alta cobertura tienen una protección elevada.

**Conclusión:**

las máscaras de tejido representan una medida preventiva de eficacia moderada en la propagación de infecciones respiratorias causadas por partículas de tamaño igual o menor al SARS-CoV-2. La eficacia de la barrera contra las gotitas se ve influida principalmente por el tipo de tejido, la cantidad de capas y la frecuencia de lavado.

## Abstract (pt)

**Objetivo:**

analisar as evidências científicas sobre a eficácia de máscaras de tecido para a prevenção da COVID-19 e outras infecções respiratórias.

**Método:**

revisão integrativa da literatura a partir da questão norteadora: “Qual a eficácia das máscaras de tecido na absorção de partículas causadoras de infecção respiratória?”. A busca ocorreu em oito bases de dados eletrônicas sem restrição de tempo e idioma.

**Resultados:**

as máscaras de tecido de baixa cobertura, 100% algodão, cachecol, fronha, fronha antimicrobiana, linho, seda, toalha de chá e saco de aspirador apresentam proteção marginal/razoável na absorção de partículas, enquanto as máscaras de tecido com alta cobertura têm elevada proteção.

**Conclusão:**

as máscaras de tecido representam medida preventiva com eficácia moderada na disseminação de infecções respiratórias ocasionadas por partículas de tamanho igual e/ou menor ao SARS-CoV-2. A eficácia da barreira contra as gotículas é influenciada, principalmente, pelo tipo de tecido, quantidade de camadas e frequência de lavagens.

## Introduction

Characterized as a pandemic by the World Health Organization (WHO), the emergent outbreak of COVID-19 has become a worldwide public health emergency^(1)^. Caused by the SARS-Cov2 coronavirus strain, the disease originated in Wuhan, China, and rapidly disseminated across countries. Given its highly transmissible nature, it has challenged the health systems and governments to urgently implement preventive measures to contain dissemination and decrease its impact^(2-3)^. In May 2020, the cases confirmed worldwide surpassed 3 million, with more than 200,000 deaths^(4)^.

With a rapid increase in cases of the disease, interpersonal contact presented itself as a risk of infection, a situation that demanded effective adherence to preventive recommendations, such as handwashing, respiratory etiquette when coughing or sneezing, wearing masks and observing social distancing. These individual and collective measures, associated with the early identification and testing of suspected cases, are essential to decrease spreading and avoid the collapse of health systems^(5-6)^.

Nonetheless, the high consumption of hospital masks on the part of the population became a problem because this piece of Personal Protective Equipment (PPE) was at risk of becoming insufficient. For this reason, the Brazilian Health Regulatory Agency (ANVISA) and the WHO recommended the population to wear non-professional masks. Thus, cloth face masks became necessary due to their preventive potential, in addition to supporting a decrease in the search for hospital masks, the priority of which should be health workers providing care to severe patients^(7-8)^.

The adoption of cloth face masks is a public health voluntary strategic measure to contain the new coronavirus. Cloth masks represent a physical barrier that may greatly impact the combat against the pandemic and significantly contribute to decreasing the incidence of COVID-19^(7)^. Hence, the number of people wearing cloth masks may interfere in the virus dissemination and flatten the disease’s growth curve, which is relevant to favor the expansion of the health system’s response capacity^(9)^.

Note that even though the use of cloth masks requires scientific proof of its efficacy in preventing the virus from spreading, the use of different types of masks coupled with hand washing and remaining preventive measures constitute a relevant strategy to decrease the dissemination of SARS-Cov2, considering the virus can rapidly spread through aerosols and droplets^(10)^.

Given this context and lack of studies addressing the efficacy of cloth face masks to prevent the new coronavirus, studies seeking evidence that support preventive measures against COVID-19 are pertinent, especially those addressing the use of cloth face masks on the part of the population, which can become co-responsible in preventing the disease. Hence, this study’s objective was to analyze scientific evidence of cloth masks’ efficacy in preventing COVID-19 and other respiratory infections.

## Method

This integrative literature review was conducted according to the following stages: identification of the study’s topic and guiding question, search for studies in the databases, critical-reflexive analysis of the studies identified, interpretation and presentation of results, and review’s final synthesis^(11)^.

Based on the Population Interest Context (PICo)^(12)^ strategy, the following guiding question was established: “How effective cloth masks are at absorbing particles that cause COVID-19 and other respiratory infections?” in which P=cloth mask; I=prevention of diseases/absorption of particles/efficacy; and Co=respiratory infections/COVID-19.

The following databases were searched: Scopus, National Library of Medicine and National Institutes of Health (PubMed/Medline), PubMed/PMC, Web of Science, Cumulative Index of Nursing and Allied Health Literature (CINAHL), Scientific Electronic Library Online (SciELO), Cochrane and Excerpta Medica dataBASE (EMBASE). To expand the results, both conventional language and descriptors were used, such as those provided by Health Science Descriptors (DECS) and Medical Subject Headings (MeSH), by crossing: (“Cloth Mask” OR “Fabric Mask” OR “Mask” OR “Face Mask”) AND “Efficacy” AND (“Respiratory Virus” OR “Influenza” OR “SARS-CoV-2” OR “Covid-19”). To fully exhaust the possibilities, the journals portal made available by the Coordination for the Improvement for Higher Education Personnel (CAPES) and accessible through the Internet Protocol (IP) coverage of the Federal University of Ceará and the State University of Acaraú was accessed.

The inclusion criterion was primary studies addressing the efficacy of cloth masks in absorbing particles. No restrictions were established for the period or language. Exclusion criteria were: dissertations, theses, literature reviews or papers not related to the study’s questions, and duplicated studies.

The process of selecting papers and verifying their eligibility followed the recommendations provided by Preferred Reporting Items for Systematic Reviews and Meta-Analyses (PRISMA)^(13)^. First, the papers’ titles and abstracts were read to select the papers that meet the inclusion criterion. Then, the studies selected were completed analyzed using a semi-structured instrument, which recorded the papers’ title, authors, year, country, methodological characteristics, and main results. Note that three independent researchers conducted the search and selected the studies to check for potential divergences.

Level of evidence was established as follows: level I referred to meta-analyses and controlled and randomized trials; level II to experimental studies; level III to quasi-experimental studies; level IV to non-experimental descriptive or qualitative studies; level V to experience reports; and level VI referred to expert opinion and consensus^(14)^.

This study complies with the ethical and legal principles provided by Resolution 510/2016, Brazilian Council of Health, concerning studies using information in the public domain.

## Results

**Figure 1:**
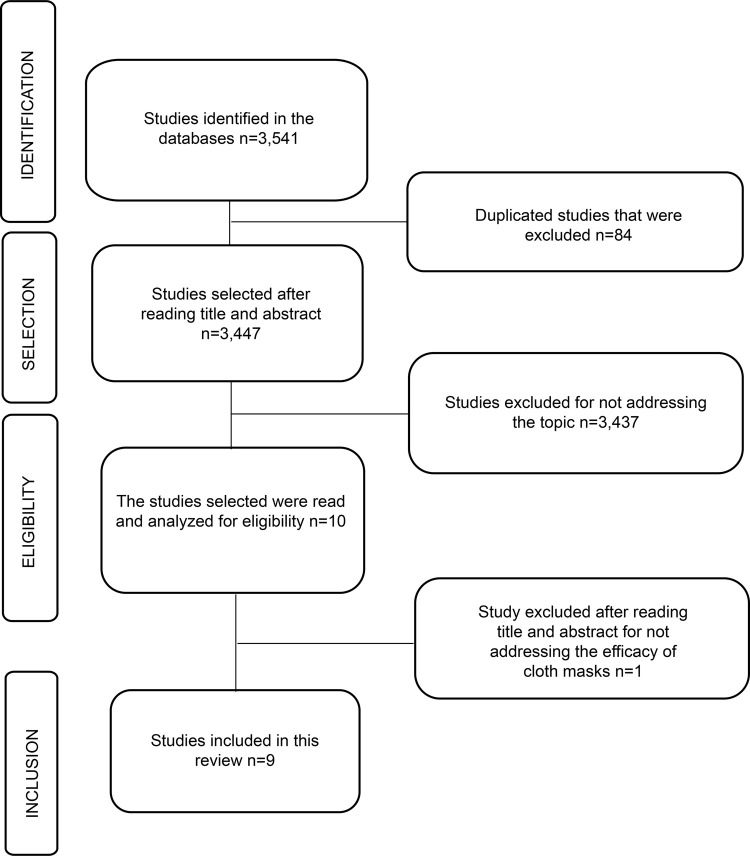
– Flowchart of the search and selection of studies according to PRISMA(13) guidelines. Fortaleza, CE, Brazil, 2020.

A total of 3,541 studies were identified, 3,447 of which were excluded for not meeting the inclusion criterion, and 84 were excluded for appearing more than once. Hence, nine studies remained in the final sample, as shown in Figure 1.

The studies dated from 2010 to 2020, most were from 2020 (44.4%). As for the studies’ country of origin, four studies were conducted in the United States (44.4%), two were from Nepal (22.2%), and one was conducted in China, Vietnam, and Portugal (11.1%), respectively. Regarding the methodological design, there was one cluster-randomized trial (11.1%), one study adopted the mathematical analysis method proposed by Kermack-McKendrick (11.1%), and seven studies adopted laboratory tests (77.7%).

**Figure 2:**
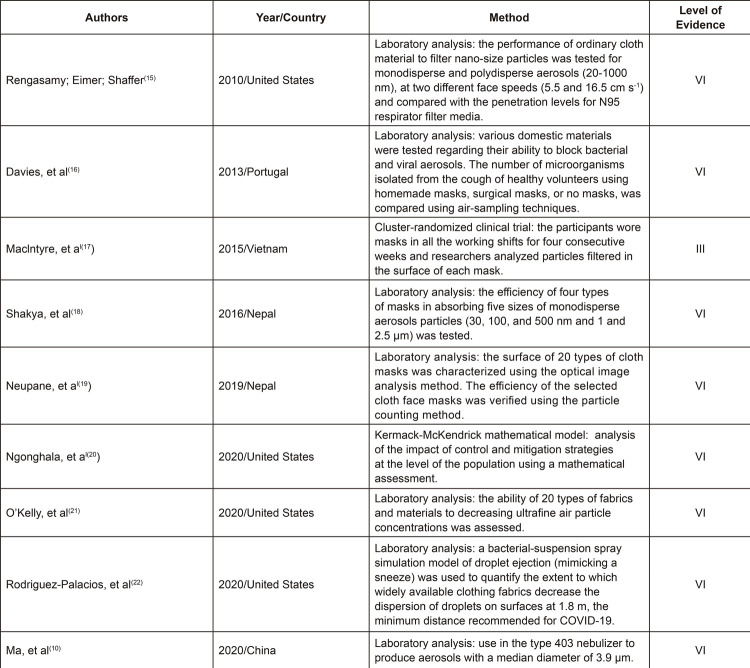
– Description of studies found in the databases according to authors, country, year of publication, and level of evidence. Fortaleza, CE, Brazil, 2020

Figure 2 presents the nine studies selected according to author, year, country, and methodological aspects.

The particles analyzed in the studies were: monodisperse and polydisperse aerosols (20-1000 nm), *Bacillus atrophaeus* (٠,٩٥-١,٢٥ μm) and *B* atrophages (23 nm), monodisperse aerosol particles (30, 100 and 500 nm and 1 and 2.5 μm), particles (<5, 5–10 and >10 μm), particles (0 to 0.8 μm), micro and macro bacteria (3x10^6-7^ cfu/ml), aerosols (median diameters of 3.9 μm and 65% of aerosols with diameters below 5.0 μm), with a frequency of 11.1% in the studies.

**Figure 3:**
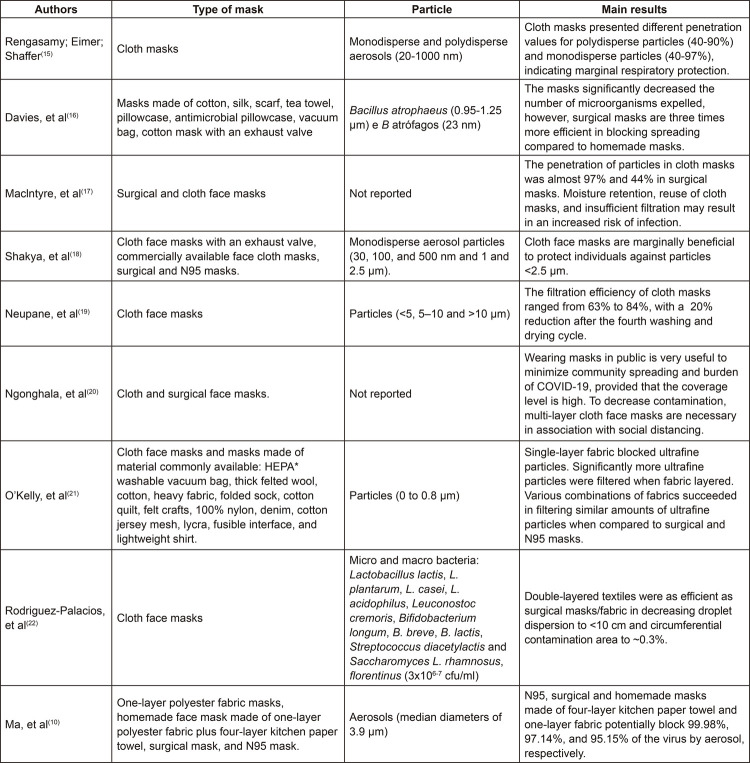
– Description of studies according to types of facemasks, samples, and main results. Fortaleza, CE, Brazil, 2020 *HEPA = High-Efficiency Particulate Arrestance

The face masks included were: masks made of cotton, silk, scarf, tea towel, pillowcase, antimicrobial pillowcase, linen, vacuum cleaner bag, of cotton fabric with an exhaust valve, High-Efficiency Particulate Arrestance (HEPA) washable vacuum bag, thick felt wool, cotton, heavy fabric, folded sock, cotton quilt, felt crafts, 100% nylon, denim, cotton jersey mesh, lycra, fusible interface, and lightweight shirt. The main results are presented in Figure 3.

Table 1 presents comparisons between types of masks and their efficacy and percentage of findings. The “low protection” efficacy level included papers reporting insufficient particle filtering; “moderate protection” included papers reporting marginal/reasonable particle filtering, and the “high protection” level included papers reporting significant particle filtering.

**Table 1:** – Type of masks, effects found, and proportion of findings among the studies. Fortaleza, CE, Brazil, 2020

Type of mask	Efficacy	Number and percentage of studies	Studies
Cloth facemask	Moderate protection	4 (80%)	Rengasamy; Eimer; Shaffer, et al ^(15)^ Shakya, et al^(18)^ Neupane, et al^(19)^ Ma, et al^(10)^
	Poor protection	1 (20%)	Maclntyre, et al^(17)^
Cloth face mask with low coverage	Moderate protection	1(100%)	Rodriguez-Palacios, et al^(22)^
Cloth face mask with high coverage	High protection	2 (100%)	Ngonghala, et al^(20)^ Rodriguez-Palacios, et al^(22)^
Masks made of cotton, silk, scarf, tea towel, pillowcase, antimicrobial pillowcase, linen, vacuum bag, mixed cotton.	Moderate protection	1 (100%)	Davies, et al^(16)^
Mask made of HEPA* washable vacuum bag, thick felt wool, cotton, heavy fabric, folded sock, cotton quilt, felt craft, 100% nylon, denim, cotton jersey mesh, lycra, fusible interface, and lightweight shirt.	High protection	1 (100%)	O’Kelly, et al^(21)^

*HEPA = High-Efficiency Particulate Arrestance

## Discussion

This study shows that most studies (44.4%) were published in 2020. Of these, one was published in China and four were published in the United States of America (USA). These results are explained by the fact that these countries represent the epicenters of the new coronavirus pandemic, which encourages researchers to develop research to fight the disease. In late April, China recorded more than 84,000 confirmed cases and more than 4,600 deaths, while the USA recorded more than one million cases and more than 60,000 deaths^(23)^.

COVID-19 is a disease caused by a positive-sense RNA virus, with 50 to 200 nm in diameter^(24)^. Studies conducted up to mid-April did not test the efficacy of masks to absorb such particles, however, there is evidence of the absorption of monodisperse and polydisperse aerosols (20-1000 nm)^(15)^, *Bacillus atrophaeus* (٠.٩٥-١.٢٥ μm) and *B* atrophages (23 nm)^(16)^, monodisperse aerosol particles (30, 100 and 500 nm and 1 and 2.5 μm)^(18)^, particles <5, 5–10 and >10 μm^(19)^, particles from 0 to 0.8 μm^(21)^, micro and macro bacteria (3x10^6-7^ cfu/ml)^(22)^, and aerosols (with median diameters of 3.9 μm)^(10)^.

Part of the studies analyzed particles smaller than those of SARS-CoV-2, as a micrometer (μm) is equivalent to 1,000 nanometers (nm). Hence, these findings may be similar to future findings regarding viral particles of coronavirus that cause COVID-19.

Additionally, a variation between 40% and 97% of protection was found among the cloth face masks addressed in the studies included in this review. This variance is related to the type of cloth used, the number of layers, and the number of washing cycles. This finding corroborates a study conducted during the outbreak of influenza A (H1N1)^(15)^, which identified that some fabrics present better filtration rates than others: towels and scarfs performed better than other cloth materials when testing monodisperse particles <100 nm (Aquis, Pinzon and Pem America). It shows that characteristics concerning the fabric fiber (diameter, load, and density) influence in the masks’ efficacy.

Studies report that the performance of cloth face masks is inferior to hospital masks (N95 and/or surgical masks); however, when double-layered, cloth masks are as efficient as hospital masks. These findings agree with the recommendations provided by the Brazilian Ministry of Health^(25)^ to contain the pandemic, as it suggests the population to make double-layered masks for own use. This was a measure of urgency taken in the process of preventing COVID-19 because personal protection equipment is scarce worldwide, and surgical and N95 masks should be saved for health workers who are more exposed to contamination by SARS-CoV-2.

In addition to Brazil, other countries have adhered to the use of homemade cloth face masks to decrease the dissemination of the COVID-19 virus, as is the case of the USA, Israel, Austria, the Czech Republic, Hong Kong, and Mongolia^(26-27)^.

As opposed to these findings, a cluster-randomized clinical trial, conducted in the wards of a hospital in Vietnam, assessed masks wore by health workers during eight-hour shifts for four weeks and verified that cotton face masks absorb almost 97% of environmental particles while surgical masks absorb 44%. Insufficient filtration is a risk for the development of infections, especially among health workers^(17)^.

In the context of a pandemic, the use of cloth face masks by the population is valid considering that scientific evidence shows its efficiency, especially when they have high coverage^(28)^. Additionally, according to the study developed in the USA, a combination of low-efficiency face masks combined with other preventive measures, especially social isolation, favor the control of the pandemic^(20)^.

As for the correct use of masks, the study conducted in Nepal shows that the efficacy of cloth masks decreases 20% after the fourth washing and drying cycle^(19)^. This decreased efficiency occurs because the cleaning process diminishes the microfibers in the fabric and increases the size of the pores. These data contradict ANVISA’s recommendations, which indicates up to 30 washing cycles^(7)^. Note that the WHO encourages the use and care of cloth masks, but does not restrict the number of washing cycles^(8)^, while the Brazilian Ministry of Health recommends changing masks after signs of wear^(25)^.

Therefore, this review presents important scientific contributions for the health and nursing fields both in the Brazilian and international contexts, because the use of cloth face masks is one of the main preventive measures recommended by health managers and health workers to contain the dissemination of the virus in the community. Hence, this study’s results provide support to strengthen the practice implemented in various countries through governmental decrees considering that part of the studies analyzed, showed moderate effectiveness in preventing respiratory infections caused by particles of similar size to SARS-CoV-2.

Note that the efficacy of the barrier provided by cloth face masks against droplets is mainly influenced by the type of fabric used, number of layers, and frequency of washings. Therefore, health workers, especially nurses, should instruct the population through social media regarding the proper use and correct washing of cloth masks to maximize and extend the protective effect of this tool for extended periods.

This study’s main limitations are related to some studies’ lack of information regarding the characteristics of the fabrics analyzed and a lack of studies addressing specific SARS-CoV-2 particles.

## Conclusion

This synthesis presents knowledge regarding nine international studies, most published in 2020, using laboratory analysis. The following nanometric and micrometric substances were studied: monodisperse and polydisperse aerosols, *Bacillus atrophaeus*, *B* atrophages, monodisperse aerosol particles, micro, and macro bacteria, and environmental and laboratory particles/aerosols. Diameters ranged from 0 μm to 1000 nm.

Low coverage cloth face masks made of 100% cotton, scarf, pillowcase, antimicrobial pillowcase, linen, tea towel, and vacuum cleaner bag presented moderate protection in the process of absorbing the particles analyzed, while high coverage cloth masks made of HEPA washable vacuum bag, thick felted wool, cotton, heavy fabric, folded sock, cotton quilt, felt crafts, 100% nylon, denim, cotton jersey mesh, lycra, fusible interface, and lightweight shirt presented high protection.

Most cloth masks presented moderate absorption of micrometric and nanometric particles so that we can infer that the filtering efficacy observed in these studies will be similar to viral particles causing COVID-19. Therefore, we believe this protective equipment handcrafted according to the recommendations provided by the health authorities of each country can contribute to the prevention of coronavirus transmission in the community, as it is a preventive measure that can favor the decrease of the disease in Brazil and the world.

We emphasize the urgency and need for further studies considering the pandemic demands the establishment of evidence-based preventive measures. While new studies are not conducted, however, we suggest the use of cloth masks is recommended to the population, especially high coverage masks (more than one layer) due to their ability to provide greater protection in absorbing nanometric and micrometric particles, similar to the SARS-CoV-2 structure. Another recommendation is to discard and replace masks after the fourth washing cycle.
